# Overexpression of transmembrane protein 168 in the mouse nucleus accumbens induces anxiety and sensorimotor gating deficit

**DOI:** 10.1371/journal.pone.0189006

**Published:** 2017-12-06

**Authors:** Kequan Fu, Yoshiaki Miyamoto, Kazuyuki Sumi, Eriko Saika, Shin-ichi Muramatsu, Kyosuke Uno, Atsumi Nitta

**Affiliations:** 1 Department of Pharmaceutical Therapy and Neuropharmacology, Faculty of Pharmaceutical Sciences, Graduate School of Medicine and Pharmaceutical Sciences, University of Toyama, Toyama, Toyama, Japan; 2 Division of Neurology, Department of Medicine, Jichi Medical University, Shimotsuke, Tochigi, Japan; 3 Center for Gene & Cell Therapy, Institute of Medical Science, The University of Tokyo, Tokyo, Japan; Tokyo Metropolitan Institute of Medical Science, JAPAN

## Abstract

Transmembrane protein 168 (TMEM168) comprises 697 amino acid residues, including some putative transmembrane domains. It is reported that TMEM168 controls methamphetamine (METH) dependence in the nucleus accumbens (NAc) of mice. Moreover, a strong link between METH dependence-induced adaptive changes in the brain and mood disorders has been evaluated. In the present study, we investigated the effects of accumbal TMEM168 in a battery of behavioral paradigms. The adeno-associated virus (AAV) *Tmem168* vector was injected into the NAc of C57BL/6J mice (NAc-TMEM mice). Subsequently, the accumbal TMEM168 mRNA was increased approximately by seven-fold when compared with the NAc-Mock mice (controls). The NAc-TMEM mice reported no change in the locomotor activity, cognitive ability, social interaction, and depression-like behaviors; however, TMEM168 overexpression enhanced anxiety in the elevated-plus maze and light/dark box test. The increased anxiety was reversed by pretreatment with the antianxiety drug diazepam (0.3 mg/kg i.p.). Moreover, the NAc-TMEM mice exhibited decreased prepulse inhibition (PPI) in the startle response test, and the induced schizophrenia-like behavior was reversed by pretreatment with the antipsychotic drug risperidone (0.01 mg/kg i.p.). Furthermore, accumbal TMEM168 overexpression decreased the basal levels of extracellular GABA in the NAc and the high K^+^ (100 mM)-stimulated GABA elevation; however, the total contents of GABA in the NAc remained unaffected. These results suggest that the TMEM168-regulated GABAergic neuronal system in the NAc might become a novel target while studying the etiology of anxiety and sensorimotor gating deficits.

## Introduction

Accumulating evidence indicates a link between the mood disorders and drug addiction within the brain’s rewarding circuitry [1, 2]. Mice chronically administered with methamphetamine (METH), are generally used as a model to learn about the psychiatric disorders. The model is described by related behavioral alterations, which suggests long-lasting influences on the gene and protein expression within specific brain subregions, including the nucleus accumbens (NAc), striatum, prefrontal cortex, and hippocampus [3–5]. Recently, several studies have attempted to elucidate the link between the METH-induced maladaptive molecular changes in the brain and behavioral alterations [4, 6, 7]. These studies may be crucial in discovering the mechanisms involved in the regulation of psychiatric phenomena and may also suggest novel targets for pharmacotherapy.

Considering using animal models of addiction to study mood disorders, we focused on the NAc, which plays an important role in both reward circuitry and mood regulation [1, 2]. Several psychostimulant adaptive molecules in the NAc are known to be involved in psychiatric disorders, including the cAMP response element-binding protein (CREB) [8, 9], brain-derived neurotrophic factor (BDNF) [10, 11], orexin [12], and Shati/Nat8l [5, 13]; however, key signaling pathways and novel molecular cascades related to behavioral regulation still remain to be identified. In a recent study, we administered METH (2 mg/kg) in mice for 6 days, and then performed a polymerase chain reaction-selected cDNA subtraction in the NAc of mice [3]. We found that a novel molecule transmembrane protein 168 (TMEM168; GenBank accession number NM_028990) was increased in the brain, especially in the NAc and hippocampus [14]. The accumbal overexpressed TMEM168 plays a crucial role in controlling the METH-induced pharmacological actions [14]; however, whether TMEM168 in the NAc is associated to the other behavioral changes in vivo still needs to be evaluated.

In the present study, the adeno-associated virus (AAV) comprising *tmem168* cDNA was microinjected into the NAc of mice to overexpress TMEM168 mRNA. A series of behavioral tests were performed to explore the behavioral changes following the interruption of the injections of TMEM168. Furthermore, the in vivo microdialysis analysis was conducted to elucidate the functional role of TMEM168 in the NAc. We identified TMEM168 in the NAc as a novel target to induce anxiety and schizophrenia-like symptoms, by inhibiting the GABAergic system in the NAc.

## Materials and methods

### Animals

Male C57BL/6J mice (8-week old; Nihon SLC, Inc. Hamamatsu, Japan) were housed in plastic cages with a 12 h light/dark cycle (8 am–8 pm). The health and welfare of the animals was monitored by staff at least once a day. All procedures were in accordance with the National Institute of Health Guideline for the Care and Use of Laboratory Animals and were approved by the Animal Experiments Committee of the University of Toyama (Permit Number A2015pha-21).

### Drugs

Diazepam (045–18901; Wako Pure Chemical Industries, Japan) was dissolved in saline and 1% Tween80. Risperidone (R3030; Sigma-Aldrich, Japan) was dissolved in saline. The behavioral experiments were performed 30 min after the drug administration. The mice administered with diazepam or risperidone were not used for other behavioral experiments.

### AAV microinjection

The AAV vector was produced according to previously described methods [15] by encoding cDNA *tmem168* (GenBank accession number NM_028990). Mice were anesthetized with a combination anesthetic (medetomidine (0.3 mg/kg), midazolam (4.0 mg/kg), and butorphanol (5.0 mg/kg)), and were fixed in a stereotactic frame (SR-5M, Narishige, Tokyo, Japan). AAV-TMEM168 vector or AAV-Mock vector (0.7 μl/side) was injected bilaterally into the NAc (1.5 mm anterior and 0.8 mm lateral from bregma, 3.9 mm below the skull surface [16]; NAc-TMEM mice or NAc-Mock mice at a speed of 0.05 μL/min. Mice were used for the experiments 3 weeks later.

All procedures were in accordance with the Guideline for Recombinant DNA Experiment from the Ministry of Education Culture, Sports, Science, and Technology, Japan and were approved by the Gene Recombination Experiment Safety Committee of the University of Toyama (Permit Number G2015pha-21).

### Quantitative real time RT-PCR analysis

After 3 weeks of AAV microinjection, the NAc-Mock mice and NAc-TMEM mice were decapitated by animal guillotine without feeling any suffering and the brains were quickly removed, since the fresh brain tissues were needed for the isolation of mRNA or brain slices. This procedure were done without anesthesia to avoid the effect of anesthetic drugs. All procedures followed the National Institute of Health Guideline for the Care and Use of Laboratory Animals (NIH publication No. 85–23, revised in 1996) and were approved by the committee for Animal Experiments of the University of Toyama (Permit Number A2015-PHA21). The NAc tissues were dissected according to the atlas of mouse brain [16] and were preserved at −80 °C until further use. The analysis of real time RT-PCR was described as a previous method [14]. The total RNA (1 μg) from each tissue was extracted (RNeasy Plus Mini Kit protocol; QIAGEN, Tokyo, Japan) and was converted into cDNA using the Prime Script RT reagent kit (Takara, Shiga, Japan), following the manufacturer’s instructions. Quantitative real time RT-PCR was performed in a Thermal Cycler Dice Real Time System (Takara) using Power SYBR Green PCR Master Mix (Applied Biosystems, Foster, CA) with cDNA and primers (1 μM), according to the manufacturer’s recommendation. The primers of TMEM168 used for real time RT-PCR were as follows: 5'-GACAGAATCATGGCATCCAAAGG-3', and 5'-ATGGACTCCAGCGGCAAGACAA-3'. The 36B4 transcript amount was quantified using primers 5'-ACCCTGAAGTGCTCGACATC-3', and 5'-AGGAAGGCCTTGACCTTTTC-3'.

### Schedule of the behavioral tests

We performed the behavioral tests in the following order: locomotor activity test, Y-maze test, novel object recognition test, social interaction test, elevated plus maze test, light/dark box test, tail suspension test, forced swim test, and prepulse inhibition test. The time interval between each test was 2–3 days.

### Locomotor activity test

The locomotor activity test was performed, as previously reported [17]. Mice were placed into a Plexiglas box with a frosting Plexiglas floor (40 × 40 × 30 cm), and the test was performed for 60 min using digital counters with infrared sensors (Scanet MV-40; MELQEST, Toyama, Japan).

### Y-maze test

Y-maze test was performed, according to a previously described method [18]. The three-arm maze (each arm measuring 40 cm × 3 cm × 12 cm) was used for the test. Mice were placed at the end of one arm and were allowed to move freely through the maze for 10 min. During this time, the arm entries were enumerated. Alternation was defined as successive entries into the three arms on the overlapping triplet sets. The percentage alternation was calculated using the following formula: (number of alternations)/(total number of arm entries-2) × 100.

### Novel object recognition test

Novel object recognition test was performed, according to a previously described method [18]. After habituation for 3 days, the NAc-Mock or NAc-TMEM mice were allowed to explore two familiar floor-fixed objects (A and B) in a Plexiglas box (30 cm × 30 cm × 35 cm) for 10 min (familiar process). The familiar object A and a novel object C were set in the Plexiglas box 24 h after the trail and the mice were allowed to explore the novel object process for 10 min (novel process). The exploratory preference percentage was calculated using the following formula: (approach time of object B or C)/(total approach time of the two objects in each process) × 100.

### Social interaction test

Social interaction test was performed according to a previously described method [19]. The apparatus for this test was designed as a Plexiglas box (60 cm × 40 cm × 22 cm) comprising three connected chambers. After habituation for 2 days, both the NAc-Mock and NAc-TMEM mice were randomly assigned to a partner male mouse, which was confined to one side of the chamber. The test mice were placed in the apparatus for 10 min and the total duration they spent interacting with the partner mouse was recorded.

### Elevated plus-maze test

Elevated plus-maze test was performed according to a previously described method [20]. The apparatus comprised four black plastic arms (25 cm × 5 cm). Two opposite arms were enclosed by walls (15 cm in height) and the other two “open” arms had only a small rim (0.2 cm) around the edges. The apparatus was elevated to a height of 70 cm above the floor level. For testing, mice were placed in the center region facing an open arm, and were allowed to freely explore the maze for 10 min. The time spent on open arms and the number of entries into the open arms was evaluated.

### Light–dark box test

Light–dark test was performed according to a previously described method [20]. The apparatus comprised two plastic chambers. The dark chamber (black plastic) measured 15 cm × 15 cm × 20 cm (l × w × h) and was covered by a lid. The light chamber, 15 cm × 15 cm × 20 cm (l × w × h), made of transparent plastic, was brightly illuminated from above with tubular fluorescent lamps (1000 lux). Mice were placed into the dark chamber and their behaviors were monitored by Scanet MV-40 LD (MELQUEST) for 10 min. The time spent in the light box was measured.

### Tail suspension test

Tail suspension test was performed according to a previously described method [19]. The mice were suspended by their tails, i.e., the body dangled in the air, with the head pointing downward. The duration of immobility from 2 min to 6 min within the 10 min test was recorded visually.

### Forced swim test

Forced swim test was performed according to a previously described method [19]. Mice were placed in a transparent Plexiglas cylinder (diameter: 14.5 cm; height: 19 cm), filled with water (depth: 15 cm; temperature: 25 °C). The immobility time was monitored by Scanet MV-40 AQ (MELQUEST) from 2 min to 6 min within the 10 min test.

### Prepulse inhibition test

Prepulse inhibition (PPI) test was performed according to a previously described method [21]. The test was evaluated using the SR-LAB apparatus (San Diego Instruments, CA, USA). Briefly, the test was performed by exposing the animals to a 70 dB background noise. After a 5 min acclimatization period, 5 pulses (120 dB each lasting 40 ms) were presented. Subsequently, the randomly prepulse plus pulse trials were administered as a 20 ms prepulse of 74, 78, 82 or 86 dB, followed by a 100 ms delay and a startle pulse (120 dB each lasting 40 ms). Eventually, 5 pulses (120 dB each lasting 40 ms) were presented once again. The PPI was calculated as (1 − [startle amplitude on prepulse + pulse trial/mean startle amplitude on pulse alone trials]) × 100.

### Tissue extraction

From each brain, the NAc tissue was bilaterally extracted and homogenized in a homogenizing buffer, containing 200 mM perchloric acid and 100 μM ethylenediaminetetraacetic acid (EDTA). The homogenates were kept in ice for 30 min and were then centrifuged at 20,000 × g for 15 min at 0 °C. Supernatant was collected and was adjusted to pH 3.0 by adding 1 M sodium acetate. After filtration (0.45 μm Membrane Filter, MF-Millipore, Japan), the extraction samples was preserved at −80 °C until the measurement by high-performance liquid chromatography (HPLC).

### In vivo microdialysis

In vivo microdialysis was performed according to a previously described method [17]. The guide cannula (AG-4, Eicom, Kyoto, Japan) was implanted into the NAc (+1.5 mm anterolateral, +0.7 mm mediolateral from bregma, and +3.25 mm dorsoventral from dura mater). On the following day, a dialysis probe (A-I-4-01, Eicom) was inserted into the guide cannula, and a ringer’s solution (147 mM NaCl, 4 mM KCl, and 2.3 mM CaCl_2_) was continuously perfused through the probe into the left side of the NAc.

In the case of GABA dialysis, the dialysate was collected every 30 min at a rate of 1.0 μL/min by a fraction collector (EF-80; Eicom), placed in biotubes and preserved at −80 °C until it was subjected to HPLC. High K⁺-stimulation (100 mM) was applied for 15 min, 4.5 h after the probe insertion. The baseline of extracellular GABA levels was the mean of the averages amount of the last three samples before high K⁺-stimulation. The 100 mM K^+^ solution means an identical amount of sodium is replaced in the ringer’s solution with potassium.

In case of dopamine and serotonin dialysis, the dialysate was collected in 15 min fractions at a rate of 0.5 μL/min and was simultaneously subjected to HPLC.

### HPLC detection

Using sampling injector (M-500; Eicom), 7 μL of o-phthalaldehyde solution (4 mmol/L) and 0.04% mercaptoethanol in carbonate buffer (pH 9.5) were added to a 21 μL of dialysate sample or extraction sample. Subsequently, 21 μL of the mixture was injected into the HPLC system (HTEC-50; Eicom). GABA was separated on the SA-50DS column (Eicom), which was maintained at 25 °C, using a phosphate buffer (pH 3.5) containing EDTA (0.5 μg/L) and 50% methanol as the mobile phase with a flow rate of 500 μL/min. An electrochemical detector that used a glassy carbon and a working electrode (set at +600 mV) against a silver–silver chloride reference electrode (WE-GC; Eicom) was used to quantify the compounds. Chromatograms were controlled by an integrator (PowerChrom: AD Instruments, NSW, Australia) connected to a personal computer.

In the case of dopamine and serotonin detection, the dialysate was injected into the HPLC system (HTEC-50; Eicom) directly by an auto injector (Eicom). Dopamine and serotonin were separated on a PP-ODS column (Eicom), which was maintained at 25 °C, using a phosphate buffer (pH 6.0) containing decane sulfonic acid (0.5 g/L), EDTA (50 μg/L), and 1% methanol as the mobile phase at a flow rate of 500 μL/min. An electrochemical detector that used a glassy carbon working electrode (set at + 400 mV) against a silver–silver chloride reference electrode (WE-3G; Eicom) was used to quantify the compounds. Four hours after the probe was inserted, the baseline of dopamine and serotonin levels was measured as the average of the last three samples. Chromatograms were controlled by an integrator (PowerChrom: AD Instruments, NSW, Australia) connected to a personal computer.

### Statistical analyses

All data were expressed as the mean ± standard error of mean (S.E.M.). Statistical differences between the two groups were determined using a Student’s *t-*test. Statistical differences among values for individual groups were determined by one-way analysis of variance (ANOVA), followed by the Bonferroni’s post hoc tests when *F* ratios were significant (*p* < 0.05). The influences of drug administration on individual groups were determined by two-way ANOVA, followed by the Bonferroni’s post hoc tests when *F* ratios were significant (*p* < 0.05). To analyze the GABA development in the microdialysis experiment, statistical differences were evaluated by ANOVA with repeated measurement, followed by Bonferroni’s post hoc tests (Prism version 5).

## Results

### Microinjection of AAV-TMEM168 vector increased the TMEM168 mRNA expression in the NAc

The mRNA expression level was measured by using real time RT-PCR experiment and was presented as the value relative to 36B4 mRNA level. The average of the TMEM168 mRNA levels in the NAc-TMEM mice was 0.214 ± 0.05 and the average of TMEM168 mRNA levels in the NAc-Mock mice (controls) was 0.0282 ± 0.003. TMEM168 mRNA levels in the NAc of the NAc-TMEM mice were increased significantly when compared with the levels in the NAc-Mock mice (*N* = 6, *p* < 0.01, *t* = 3.979; Student-*t* test).

### Overexpression of TMEM168 did not change the locomotion, spontaneous alternation, cognitive ability, social interaction, and depression-like behaviors in mice

**Fig 1:**
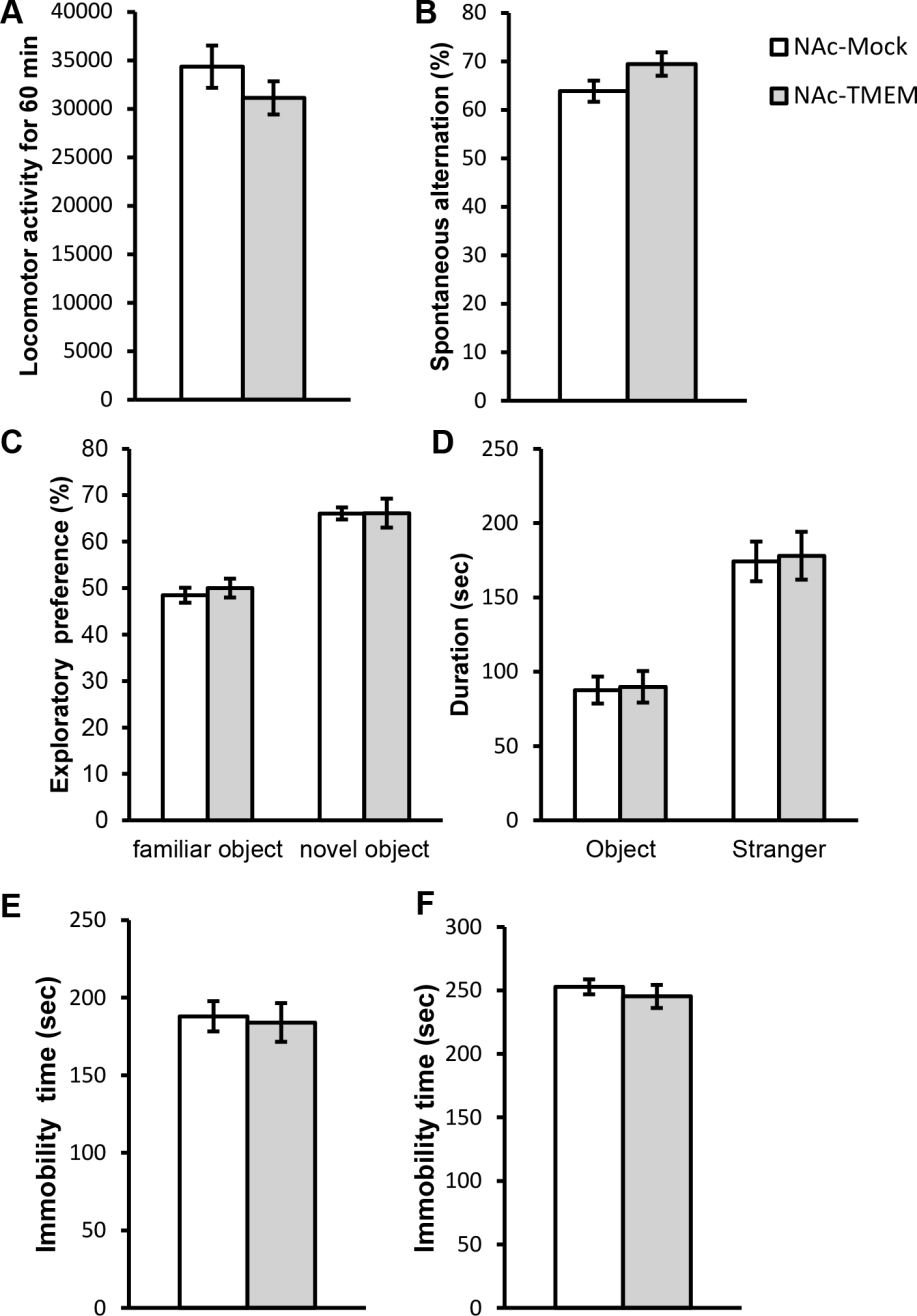
Similar preferences in locomotor activity, Y-maze, novel object recognition, three chambers, tail suspension, and forced swimming tasks in the NAc-TMEM mice compared with the NAc-Mock mice. (A) The locomotor activity in the NAc-Mock and NAc-TMEM mice were measured for 60 min (No significant difference; Student-*t* test). (B) Working memory was assessed in the Y-maze spontaneous alternation task in the NAc-Mock and NAc-TMEM mice (No significant difference; Student-*t* test). (C) Cognitive function was assessed in the novel object recognition task. Percentage of total exploratory time on the novel object was expressed as exploratory preference (%) (No significant difference; ANOVA followed by the Bonferroni’s post hoc tests). (D) Social interaction was assessed in the three chambers task. Average time (10 min per phase) spent in the chamber with an object or a stranger mouse was detected (No significant difference; ANOVA followed by the Bonferroni’s post hoc tests). (E) Immobility time of the NAc–-Mock and NAc-TMEM mice in the tail-suspension task was measured for 5 min (No significant difference; Student-*t* test). (F) Immobility time of the NAc-Mock and NAc-TMEM mice in the forced swimming task was measured for 5 min (No significant difference; Student-*t* test). Values are presented as mean ± S.E.M. N = 9.

A series of behavioral tests were performed to detect the changes in the emotional behavior induced by TMEM168 overexpression. The NAc-TMEM mice reported no changes in the locomotor activity test (Fig 1A, *t* = 1.167) or the Y-maze test (Fig 1B, *t* = 0.9495), novel object recognition test (Fig 1C, *F*_(3, 32)_ = 20.98), three chamber social interaction test (Fig 1D, *F*_(3, 32)_ = 15.7), tail suspension test (Fig 1E, *t* = 0.2432), and forced swimming test (Fig 1F, *t* = 0.7084) when compared with the NAc-Mock mice.

### Overexpression of TMEM168 in the NAc induced the increased anxiety and decreased sensorimotor gating in mice

**Fig 2:**
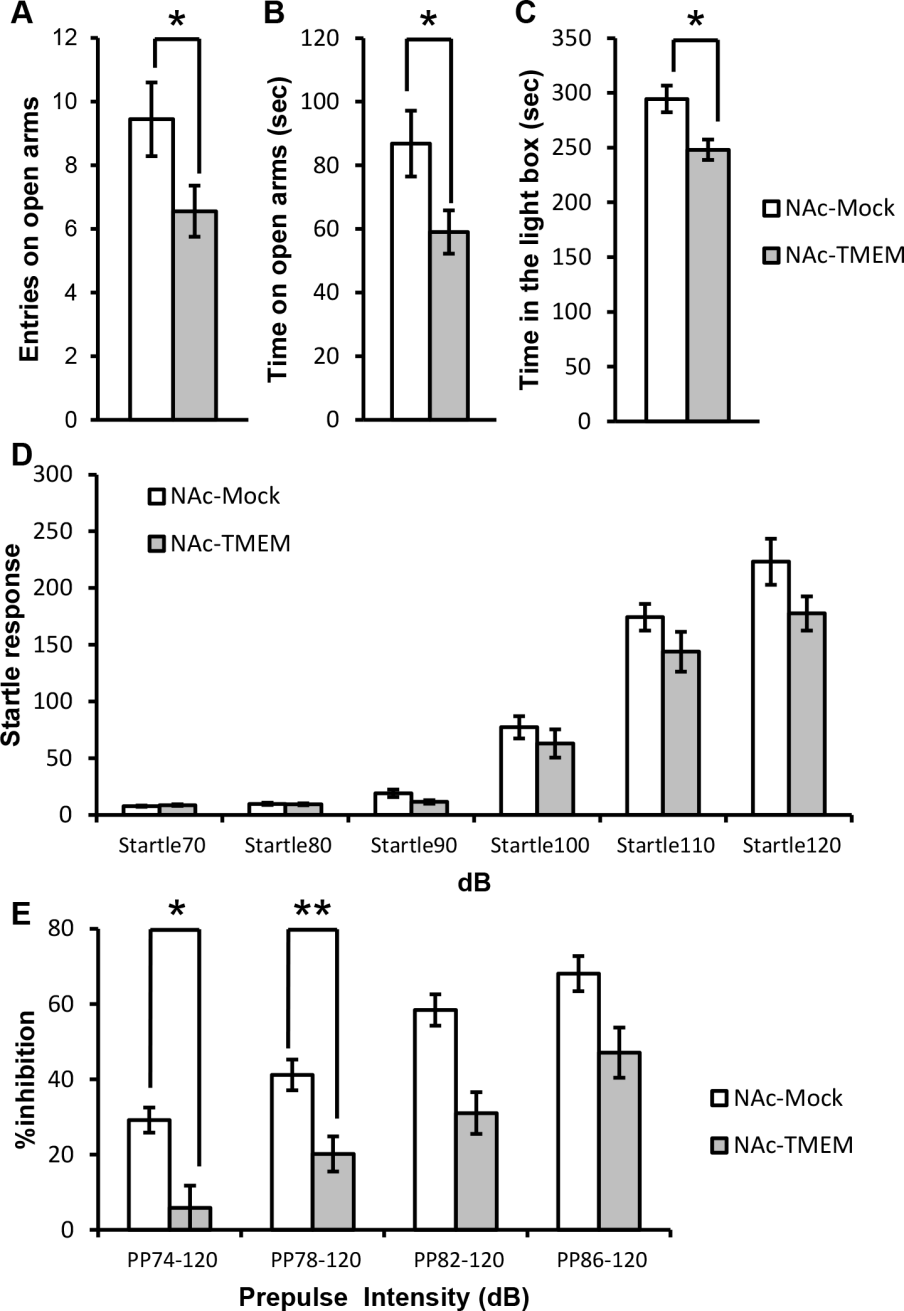
Increased anxiety and decreased PPI in the NAc-TMEM mice compared with the NAc-Mock mice. (A) Number of entries spent on open arms were measured for 10 min in the elevated plus-maze task; *N* = 9; Values are presented as mean ± S.E.M. *p < 0.05 vs. NAc-Mock (Student-*t* test). (B) Time spent on open arms was measured for 10 min in the elevated plus-maze task; *N* = 9; Values are presented as mean ± S.E.M. *p < 0.05 vs. NAc-Mock (Student-*t* test). (C) Time in the light box was measured for 10 min in the light/dark box task; N = 9; Values are presented as mean ± S.E.M. *p < 0.05 vs. NAc-Mock (Student-*t* test). (D) Startle responses were measured at 70, 80, 90, 100, 110, and 120 dB, respectively (background noise: 70 dB). *N* = 9; Values are presented as mean ± S.E.M. No significant difference between NAc-TMEM and NAc-Mock mice (ANOVA followed by the Bonferroni’s post hoc tests). (E) PPI was measured for 74, 78, 82, and 86 dB, respectively, of the prepulse intensity (background noise: 70 dB). Values are presented as mean ± S.E.M. *N* = 9. *p < 0.05 vs NAc-Mock (ANOVA followed by the Bonferroni’s post hoc tests).

TMEM168 overexpression in the NAc increased anxiety in mice, such as entries (Fig 2A, *p* < 0.05, *t* = 2.844) and time (Fig 2B, *p* < 0.05, *t* = 2.2.253) on open arms in the elevated plus-maze) as well as time in the light box in light/dark box tasks (Fig 2C, *p* < 0.05, *t* = 2.964). Although the startle responses were not affected (Fig 2D, *F*_(11, 96)_ = 58.07), decreased sensorimotor gating in the NAc-TMEM mice was observed in the prepulse intensity of 74 dB and 82 dB in the auditory PPI test (Fig 2E, *p* < 0.05, *F*_(7, 64)_ = 16.61). It is suggested that overexpression of TMEM168 in the NAc induced sensorimotor gating deficit in mice.

### Anxiety-like behaviors induced by TMEM168 overexpression in the NAc were reversed by the administration of diazepam

**Fig 3:**
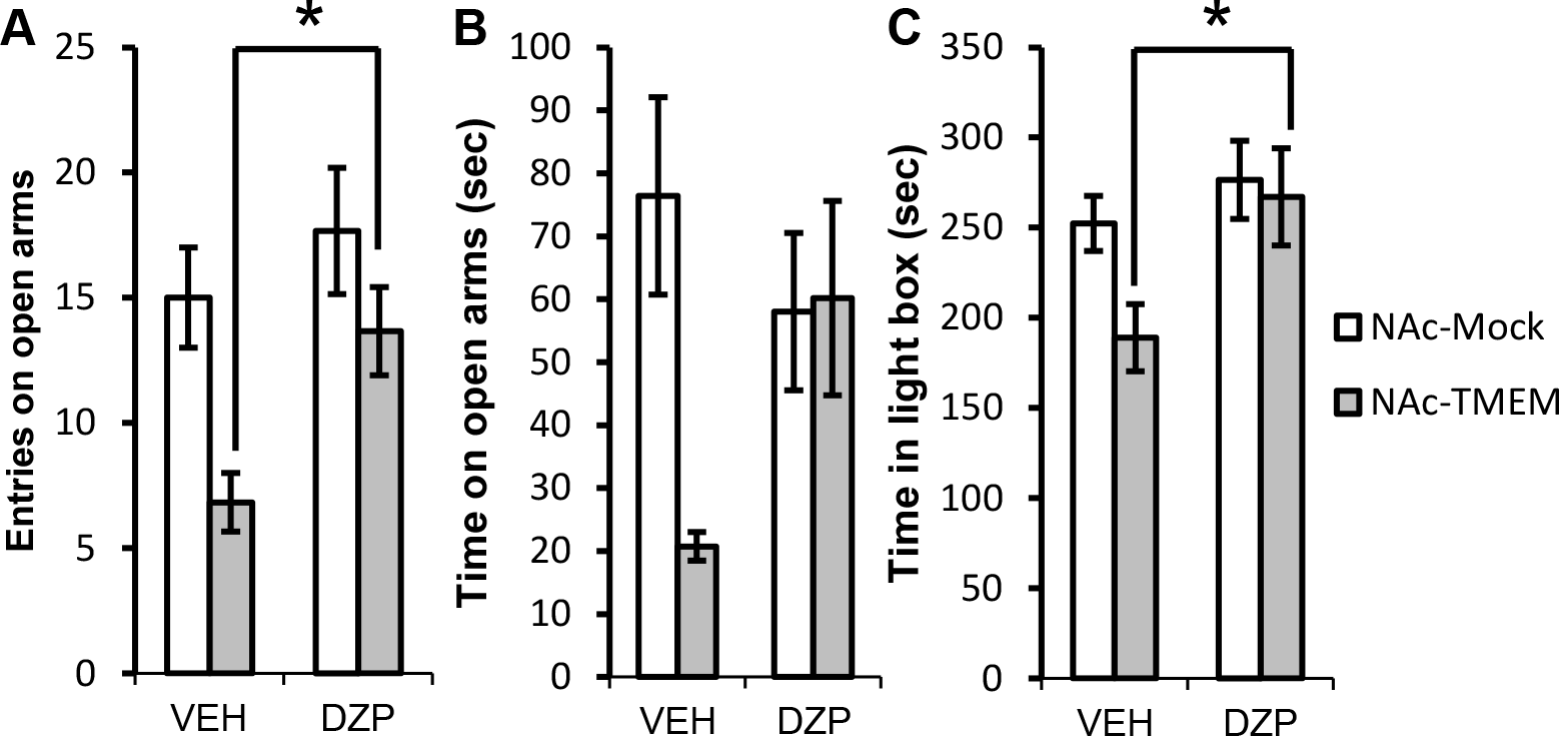
Reversal of anxiety behaviors in the elevated plus-maze and light/dark box task following the administration of diazepam in the NAc-TMEM mice. (A) and (B) Diazepam (0.3 mg/kg i.p.) or vehicle was administered 30 min before performance in the elevated plus-maze task. Number of entries and time on open arms were measured for 10 min in the elevated plus-maze task, *N* = 6; Values are presented as mean ± S.E.M. *p < 0.05 vs. NAc-TMEM (VEH) (two-way ANOVA followed by the Bonferroni’s post hoc tests). (C) Diazepam (0.3 mg/kg i.p.) or vehicle was administered 30 min before the light/dark box test. Time in the light box was measured for 10 min in the light/dark box test, *N* = 8; values are presented as mean ± S.E.M. *p < 0.05 vs. NAc-TMEM (VEH) (two-way ANOVA followed by the Bonferroni’s post hoc tests); VEH: vehicle administration group, DZP: diazepam administration group.

Diazepam is an (Food and Drug Administration in USA (FDA)-approved benzodiazepine known to alleviate anxiety, by activating the inotropic GABA_A_ receptors [22]. To investigate whether the anxiety-like behaviors detected in the NAc-TMEM mice could be reduced by the administration of anxiolitic drugs, mice were administered with diazepam (0.3 mg/kg i.p.) or vehicle, 30 min before a performance in the elevated plus-maze and the light/dark box tasks. The dose of diazepam for mice administration was referred to the previous study [23], which would not affect anxious behaviors in mice as a criticality. In the elevated plus-maze tasks, the decreased number of open arm entries in the TMEM mice was reversed (Fig 3A, *F*_(1, 20)_ = 1.169, *p* < 0.05) and the decreased time spent in open arms tend to be normalized in the NAc-TMEM mice (Fig 3B, *F*_(1, 20)_ = 5.2), following the administration of diazepam. Similarly, in the light/dark box task, the decreased time spent in the light box in the NAc-TMEM mice was also reversed after the administration of diazepam (Fig 3C, *F*_(1, 28)_ = 1.628, *p* < 0.05).

### Decreased PPI induced by TMEM168 overexpression in the NAc was reversed following the administration of risperidone

**Fig 4:**
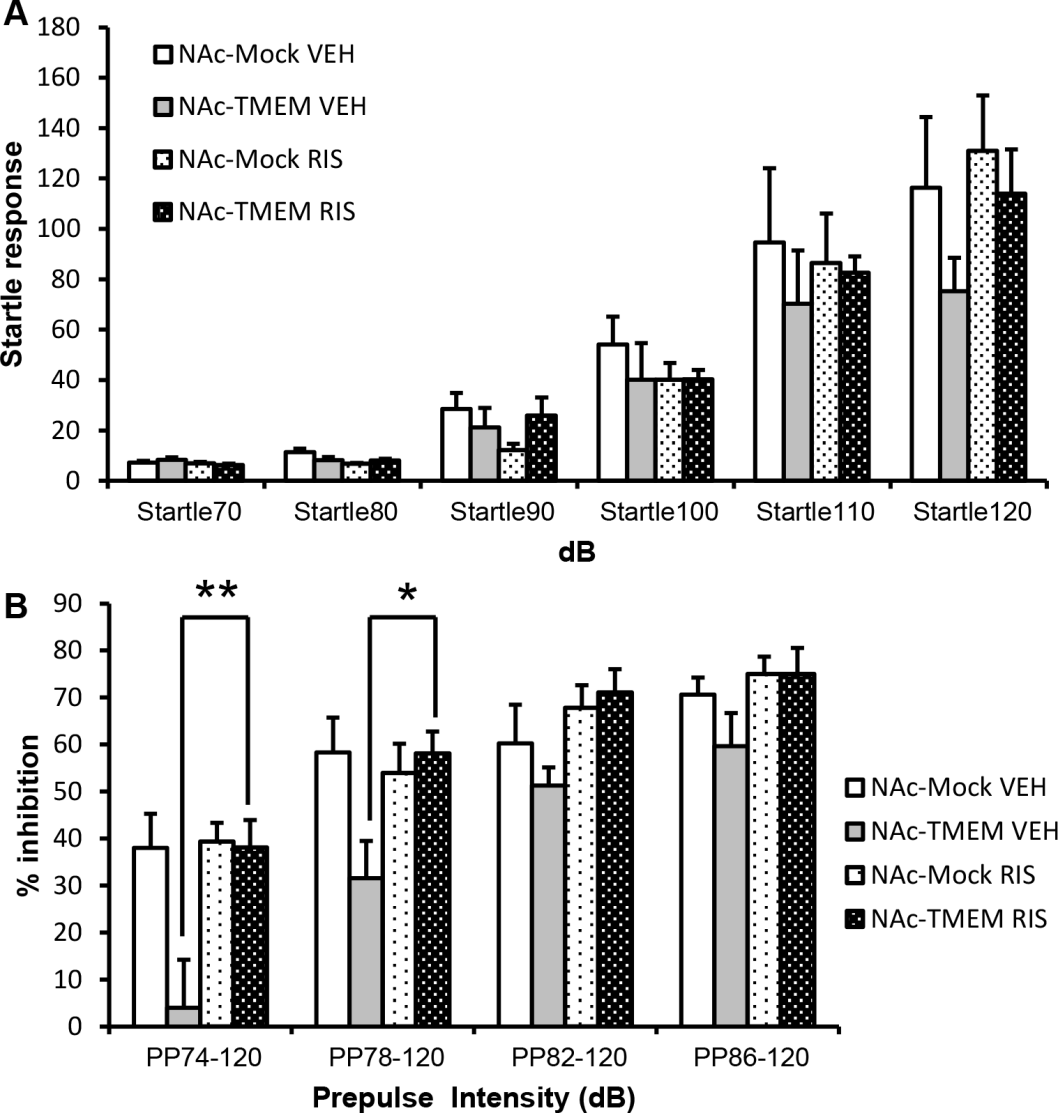
Reversal of sensorimotor gating deficit in the PPI task following the administration of risperidone in the NAc-TMEM mice. (A) Risperidone (0.01 mg/kg i.p.) or saline was administrated 30 min before the task performance. Startle responses was measured at 70, 80, 90, 100, 110, and 120 dB, respectively (background noise: 70 dB), *N* = 8; values are presented as mean ± S.E.M. No significant difference between NAc-TMEM and NAc-Mock mice (two-way ANOVA followed by the Bonferroni’s post hoc tests). (B) Risperidone (0.01 mg/kg i.p.) or saline was administered 30 min before the task performance. PPI was measured for 74, 78, 82, and 86 dB respectively, of prepulse intensity (background noise: 70 dB), *N* = 8; values are presented as mean ± S.E.M. **p < 0.01, *p < 0.05 vs. NAc-TMEM (VEH) (two-way ANOVA followed by the Bonferroni’s post hoc tests). VEH: saline administration group, RIS: risperidone administration group.

Sensorimotor gating deficit, which is detected by auditory PPI test, is assumed to be a distinctive phenomenon of schizophrenia [24]. Previous studies reported that antipsychotic drugs, such as risperidone, significantly reverse the low levels of sensorimotor gating [25]. Mice were injected with risperidone (0.1 mg/kg i.p.) or saline, 30 min before performing the auditory PPI task. The concentration of risperidone administration was referred to the previous studies [26, 27], which would not affect locomotor activity and startle response in mice. No between-group difference was observed in the startle response to any pulse intensity between the NAc-Mock mice and NAc-TMEM mice, when these were administrated with saline or risperidone (Fig 4A, *F*_(11, 162)_ = 0.6238). However, the decreased PPI in the NAc-TMEM mice was reversed following the administration of risperidone at a prepulse of 74 dB (*p* < 0.01) and 78 dB (*p* < 0.05) (Fig 4B, *F*_(7, 108)_ = 2.293).

### Overexpression of TMEM168 in the NAc did not change the total contents of glutamate and GABA, but decreased the basal levels of accumbal extracellular GABA and high Kζ-stimulated GABA release from the NAc

**Fig 5:**
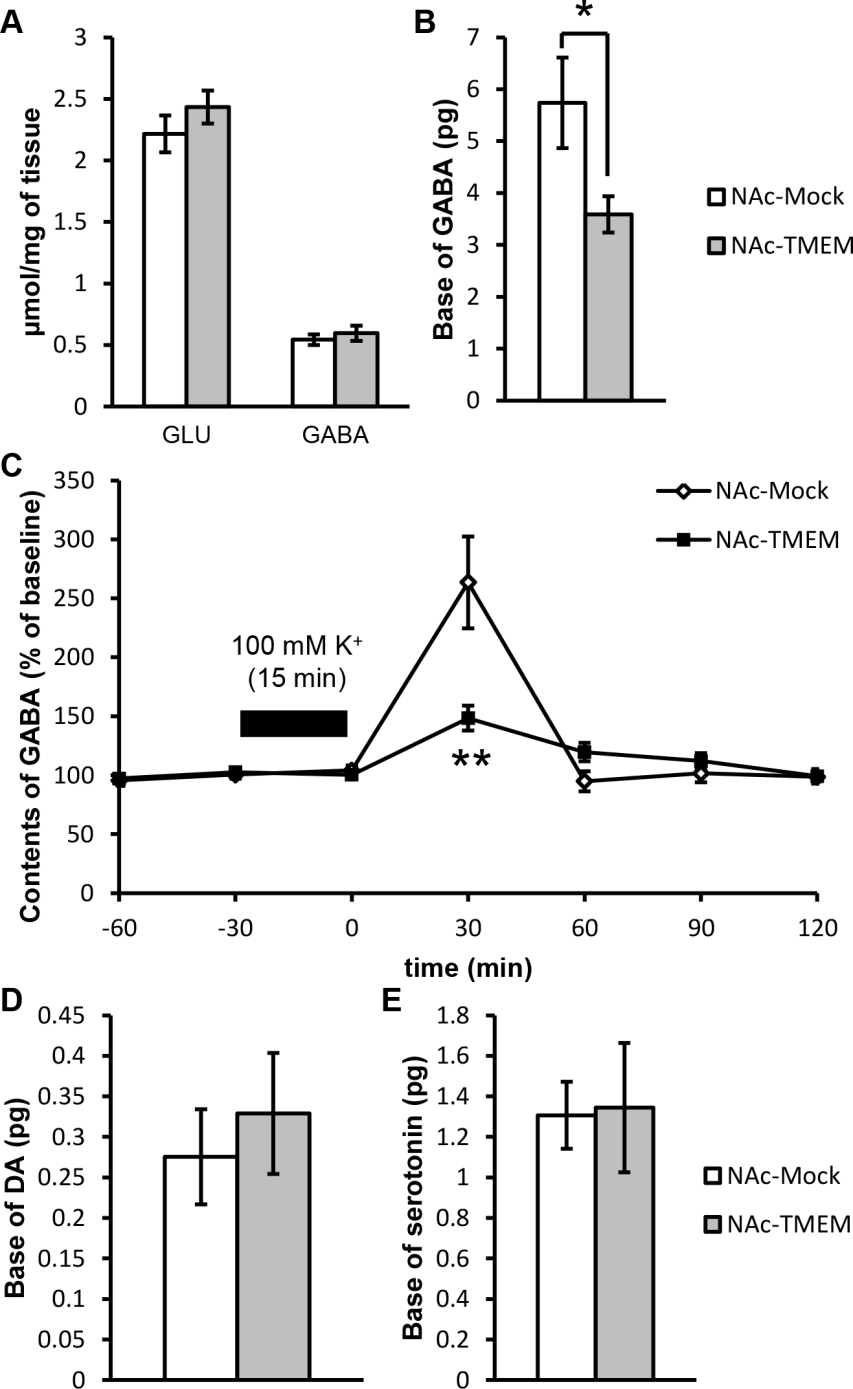
Inhibitory effects of TMEM168 on GABA neurotransmission in the NAc. (A) Glutamate (GLU) and GABA concentrations in the NAc tissue were measured by HPLC. The NAc-Mock mice and NAc-TMEM mice were sacrificed and then the NAc tissue was extracted immediately, *N* = 11; values are presented as mean ± S.E.M. No significant difference between NAc-TMEM and NAc-Mock mice (two-way ANOVA followed by the Bonferroni’s post hoc tests). (B) Basal levels of extracellular GABA in the NAc were detected by the in vivo microdialysis task, *N* = 6; values are presented as mean ± S.E.M. *p < 0.05 vs. NAc-Mock (Student-*t* test). (C) Dynamic changes in the extracellular GABA levels in the NAc after high K⁺-stimulation was analyzed in the NAc by the in vivo microdialysis task, *N* = 6; values are given as mean ± S.E.M. **p < 0.01 vs. NAc-Mock. (ANOVA with repeated measures followed by the Bonferroni’s post hoc test) (D) Basal levels of extracellular dopamine in the NAc were detected by the in vivo microdialysis task, *N* = 4; values are presented as mean ± S.E.M. No significant difference between NAc-TMEM and NAc-Mock mice (Student-*t* test). (E) Basal levels of extracellular serotonin in the NAc were detected by the in vivo microdialysis task, *N* = 3–4; values are given as mean ± S.E.M. No significant difference between NAc-TMEM and NAc-Mock mice (Student-*t* test).

The contents of GABA and glutamate in the NAc were analyzed by HPLC. No difference was observed between the NAc-TMEM and NAc-Mock mice (Fig 5A, *F*_(1, 40)_ = 0.5878). The TMEM168 overexpression inhibited the basal extracellular GABA levels (Fig 5B, *p* < 0.05, *t* = 2.281). Moreover, GABA release following the potassium stimulation was decreased in the NAc-TMEM mice when compared to the NAc-Mock animals (Fig 5C, *p* < 0.01, *F*_(6, 60)_ = 7.683). These results suggest that TMEM168 baseline overexpression attenuated GABA neurotransmission in the NAc.

### Overexpression of TMEM168 did not change the basal amount of extracellular dopamine and serotonin in the NAc

The basal levels of accumbal extracellular dopamine (Fig 5D, *t* = 0.5635) and serotonin (Fig 5E, *t* = 0.09495) in the NAc-Mock and NAc-TMEM mice were analyzed using the in vivo microdialysis method. No significant between-group difference was observed.

## Discussion

Both sensorimotor gating deficit and increased anxiety are often found in patients with addiction disorder [4, 28]. Repeated METH administration in rodents is usually used as a model to mimic the decreased PPI and anxiety in schizophrenia [21, 29, 30]; however, a clear link between METH addiction and emotional properties or sensorimotor gating function still needs to be assessed. In the present study, we found that the increased METH related molecule TMEM168 in the nucleus accumbens, induced anxiety in the elevated plus-maze and light/dark box tasks, and resulted in sensorimotor gating deficit in the auditory PPI task. These findings suggest that TMEM168 in the NAc is crucial for the modulation of anxiety and schizophrenia-like behaviors in mice.

GABA is a primary inhibitory neurotransmitter associated with emotion regulation anomalies, including anxiety and panic disorders [31]. Specifically, the reduced concentration of GABA is thought to be associated with increased anxiety levels [31]. As the injected AAV-TMEM168 vector can transduce into local neurons preferentially [32, 33], approximately 99% of the affected neuronal populations in the NAc of NAc-TMEM mice should be GABA neurons [2, 34]. In vivo microdialysis analysis revealed that the basal levels of extracellular GABA were reduced in the NAc, and GABA release was also reduced after K^+^ stimulation in the NAc-TMEM mice when compared with the control mice. Furthermore, the pharmacological action of anxiety reducing drug, diazepam, which is known to facilitate GABAergic transmission by binding GABA_A_ receptors [22], reversed the TMEM168 overexpression-induced anxiety as measured in both the elevated plus-maze and light/dark box tasks. These results suggest that a reduction in GABAergic neurotransmission could be linked to TMEM168-induced anxious behaviors.

The trigger of anxiety is a complex process in the brain, which is related to the activity in multiple neural circuits. Briefly, the amygdala, bed nucleus of the stria terminalis, and prefrontal cortex (PFC) are usually identified as the key regions controlling anxiety. As a central relay structure between the amygdala, basal ganglia, ventral tegmental area (VTA), and PFC, the NAc seems to play a modulatory role in the anxious signal transmission from the amygdaloid complex to the latter areas [35]. In the present study, we found that GABA release was inhibited following a TMEM168 transfection in the NAc neurons locally, including 95% GABAergic medium spiny neurons (MSN) projecting to other brain regions [2, 34]. As the direct projected targets of the accumbal MSN, the VTA and pallidum are demonstrated to be relevant to anxiety symptoms via GABAergic dysfunction [36, 37]. Thus, the interrupted GABAergic projection from the NAc might underlie the mechanism of the increased anxiety in the NAc-TMEM mice.

The NAc-TMEM mice also showed reduced PPI when compared with the NAc-Mock mice in the present auditory startle response test. Increased dopaminergic and serotoninergic neurotransmission in the brain is presumed to reduce PPI in rodents [38, 39]. Risperidone is an antagonist of dopamine receptor D2, and serotonin receptor 2A in multiple brain regions [40]. In the present study, risperidone reversed the sensorimotor gating deficit associated with the overexpression of TMEM168 in the NAc. This might indicate that the overexpression of TMEM168 in the NAc could mediate sensorimotor deficits through an increase of dopaminergic or serotoninergic activity. However, no significant difference in accumbal extracellular dopamine or serotonin between the NAc-TMEM and NAc-Mock mice was observed. Numerous animal and human studies have indicated that sensorimotor gating function is regulated by the cortico-striatal-pallido circuit [24, 41]. Hence, the interruption in the NAc might not be a solitary part of the integral neural pathways. There is a possibility that the dopaminergic and serotoninergic functions in other accumbal relevant regions such as the PFC, striatum, and pallidum are indirectly affected by the GABAergic suppression in the NAc, and their dysfunctions are subsequently normalized by the administration of risperidone in the NAc-TMEM mice. Although the neurotransmissions in these accumbal relevant regions of the NAc-TMEM mice are needed to be analyzed in the next study, the functional roles of accumbal TMEM168 in the METH-induced schizophrenia-like behaviors were demonstrated firstly in the present experiment. As TMEM168 is an adaptive molecule responding to METH exposure, the study of the increased TMEM168 in the NAc might open a branch to elucidate the mechanism of the METH-induced psychotic complications, of which one characteristic symptom is sensorimotor gating deficit.

The downstream signaling pathways of TMEM168 in influencing GABAergic activity or behavioral events still remain unclear. Repeated administration of METH does not influence the extracellular GABA levels in the NAc, but the overexpression of TMEM168 via the AAV vector transfection inhibits the accumbal GABA release. It is suggested that TMEM168 may play some functional roles in GABAergic regulation independent on the pharmacological effect of METH. The Crk-like protein (CrkL), for example, has been found to interact with TMEM168 in a yeast two-hybrid screening study [42]. CrkL, collectively with Crk, participates in the reelin signaling cascade downstream of DAB1 [43, 44]. The reduced expression of reelin can weaken the GABAergic neurotransmission in transgenic mice and also schizophrenia or bipolar patients [45–48]. Thus, it could be suggested that the activation of the TMEM168-CrkL-reelin pathway might induce behavioral changes in the NAc-TMEM mice altering the GABAergic neurotransmission. Furthermore, in a previous study, we found that extracellular osteopontin (OPN) was increased in the NAc-TMEM mice [14]. Activation of integrin receptors is usually determined as the downstream signaling pathway of the secreted OPN [49]. Mutations of β1- and β3-containing integrins in mice have been linked to anxiety disorders [50]. Thus, the TMEM168-OPN-integrin receptor could also be implicated in the mechanisms underpinning TMEM168-effects on behavior.

In summary, TMEM168 overexpression in the NAc neurons could induce a decrease in the extracellular GABA levels in the NAc, with effects on both anxiety levels and sensorimotor gating ability. Future research should further explore the role of TMEM168 in emotional properties or sensorimotor gating function.
